# Color Stability, Gloss Retention, and Surface Roughness of 3D-Printed versus Indirect Prefabricated Veneers

**DOI:** 10.3390/jfb14100492

**Published:** 2023-09-28

**Authors:** Arwa Daghrery

**Affiliations:** Department of Restorative Dental Sciences, College of Dentistry, Jazan University, Jazan 45142, Saudi Arabia; ardaghrery@jazanu.edu.sa

**Keywords:** printing, artificial aging, color, gloss, roughness, veneers

## Abstract

The long-term color stability and surface properties of anterior laminate veneers are among the crucial factors affecting the clinical longevity of aesthetic restorations. Novel 3D-printed materials are being introduced as definitive restorative treatment. In light of the existing variety of indirect yet minimally invasive composite resin veneers, research on their surface properties is warranted. This in vitro study evaluated the effect of artificial aging by immersion in different staining solutions on the color changes, gloss, and surface roughness (Ra) of 3D-printed veneers compared to the prefabricated resin composite veneer systems (PRCVs) manufactured by Componeer and Edelweiss. Moreover, this study compared the effects of two methods for stain removal: repolishing with Sof-Lex disks and in-office bleaching with 40% hydrogen peroxide. The veneers (*n* = 24) were randomly divided according to the immersion solutions used, i.e., tea and coffee. Colorimetric measurements, surface roughness, and surface gloss were determined before and after staining and surface treatment with either in-office bleaching or surface polishing. The data were statistically analyzed using two-way ANOVA followed by the Tukey’s post hoc test (α = 0.05). Artificial aging with immersion in staining solutions led to significant color changes, increased surface roughness, and gloss reduction in all materials (*p*  <  0.05). The 3D-printed veneers showed higher Δ*E* values (coffee = 10.112 ± 0.141) and (tea = 10.689 ± 0.771) compared to baseline after 7 days of aging. The 3D-printed veneers had a statistically significant surface roughness Ra (0.574 µm ± 0.073). The gloss was >70% in all groups at baseline; these values dropped in all groups after 7 days of artificial aging. After the stain-removing procedures, the Δ*E* values decreased in all tested veneers. That being said, they failed to return to the baseline values, and both stain-removing methods were found to have an adverse effect on surface roughness and gloss retention in all tested veneers.

## 1. Introduction

The increasing demand for aesthetic restorative treatments and recent advances in manufacturing technologies have led to the development of tools and biomaterials to restore the natural appearance of anterior teeth. Hence, anterior laminate veneers offer a minimally invasive alternative to traditional treatments for closing small diastemas, correcting misshapen teeth, or improving the color of discolored teeth [1]. For decades, ceramic veneers have been popular for veneer manufacturing due to their superior aesthetic properties, satisfactory abrasion resistance, enhanced biocompatibility, and potential color stability [2]. Nevertheless, the demand for affordable, minimally invasive, time- and cost-saving manufacturing of aesthetic restorations has increased [3].

Therefore, numerous new indirect restorative materials are currently available on the market, each with distinct and essential chemical, physical, and mechanical properties. These properties can undoubtedly affect clinical survival, success rates, aesthetic results, and costs, thus making them a crucial consideration for any dental practitioner [4]. Over the past years, advances in materials, digital tools, and additive manufacturing (AM) technologies have improved clinical outcomes and restoration longevity. Among AM technologies, 3D printing enables the manufacture of high-resolution restorations with adequate surface finish. It reduces the manufacturing time and minimizes wastage of raw material; thus, it can be a highly cost-effective option for fabricating long-term provisional to definitive restoration.

To meet the demand for economical and user-friendly alternatives without compromising mechanical and physical properties, 3D-printed veneers have been developed [5]. These veneers utilize nanohybrid composite resin materials, which are recommended as the definitive restorative materials [5,6]. Furthermore, a parallel advancement in resin-based veneers was the advent of a new generation of prefabricated resin composite veneers (PRCVs) [7,8]. The PRCVs are laboratory-fabricated composite veneers with superior wear resistance, fracture toughness, and exceptional aesthetics [9]. The introduced veneer manufacturing techniques, such as laser vitrification and heat and pressure molding, have rejuvenated the usefulness of these veneer systems [8]. Resin-based veneers combine the aesthetics of ceramic surfaces and bondability to tooth structure [10]. Hence, composite veneers establish themselves as a more conservative veneering modality [10]. 

Though the 3D-printed veneers and PRCVs provide minimally invasive indirect restorative solutions for managing anterior teeth, the 3D-printed veneers deliver on-demand chairside treatment options with higher accuracy and are designed based on preparation dimension. Importantly, using 3D printing technology for manufacturing dental prostheses reduces waste and enables simultaneous processing of multiple prostheses [11].

Notably, the development of dental materials is an intricate process that encompasses multiple fields of knowledge. One crucial aspect is comprehending how the challenging oral environment and function impact restorative materials’ clinical efficacy. Although composite resins are renowned for their superior aesthetics, their vulnerability to discoloration in oral environments is a significant drawback [12,13]. 

The color stability of restorative materials is influenced by various factors such as exposure to different types of stains, the composition and surface roughness of the material, and the duration of exposure [14]. Moreover, the manufacturing technique can also impact a restorative material’s color stability [15]. For instance, 3D printing technology may cause stair-stepping phenomena, especially on curved surfaces, which can significantly affect the color stability and aesthetics of 3D-printed resin materials used for anterior aesthetic restorations [5]. Moreover, the post-polymerization procedure used as part of the AM technique can also directly affect the color stability of the printed materials by influencing its final structure [5,16].

There have been various methods suggested for stain removal from resin restorative materials [14,17]. Studies have indicated that in-office bleaching is a less invasive approach to removing stains compared to surface polishing [17]. Nevertheless, the success of the bleaching method in eliminating stains varies based on the type of stain and the composition of the restorative materials [18].

To that end, two aims were developed: *i*. to compare the 3D-printed veneers to the PRCVs in terms of color changes, gloss retention, and surface roughness after artificial aging and *ii*. to evaluate the effects of bleaching and repolishing on the color stability, gloss retention, and surface roughness of the 3D-printed veneers compared to the PRCV systems. The null hypotheses were as follows: (1) The type of manufacturing technique does not affect the discoloration tendency of the tested veneer materials. (2) The type of manufacturing technique does not affect the gloss retention and surface roughness of the tested veneer materials. (3) Stain removal modalities such as bleaching and surface repolishing do not affect the color stability, gloss retention, and surface roughness of the tested veneers.

## 2. Materials and Methods

### 2.1. Samples Preparation

The PRCVs were obtained directly from the manufacturer’s C-PRCV systems (Componeer Brillian, Coltene, Altstätten, Switzerland) and E-PRCVs (Edelweiss, Wolfurt, Austria) (Table 1). Both PRCVs are prefabricated veneers made of pre-polymerized highly filled nanohybrid composite resin. Then, one PRCV of an upper central incisor was scanned using optical scanner (MD-ID0300, Medit, Seoul, Korea). The exported STL file was opened on a Dfab Chairside 3D blue edge laser printer (DWS, Thiene, Italy) with the TSLA (tilted stereolithography) technology. The print base and the disposable platform (measuring 5 × 2 cm) were introduced into the Dfab. Then, a disposable DWS Irix Max, a recently introduced nanohybrid composite resin material, cartridge with ceramic fillers (42% by weight) incorporating the vat was also inserted into the Dfab. An A2 monochrome cartridge was used. After visual inspection, a wash in 95% ethyl alcohol was performed to remove liquid composite and clean the veneers. The veneers were then removed from the supporting sprues (which have a patented design that avoids any damage to the connected surface, allowing for a clean and secure separation) and post-cured in an ultraviolet light-curing unit (Dcure, DWS, Thiene, Italy) for 9 min, per the manufacturer’s instructions. The 3D-printed veneers were polished using a series of Soflex^®^ discs (SofLex, 3M Espe, Saint Paul, MN, USA), following previously reported protocol [15].

**Figure 1:**
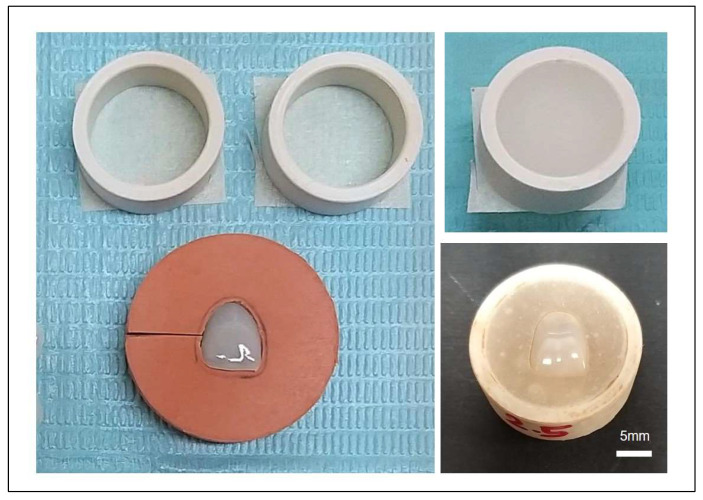
Illustration of sample preparation for experiments.

All the veneers in each group (*n* = 24 C-PRCVs, *n* = 24 E-PRCVs, and *n* = 24 3D-printed veneers) underwent a thorough cleaning process using distilled water in an ultrasonic cleaner (Branson CPX1800H Ultrasonic Cleaner⁄Branson Inc., Danbury, CT, USA). The tested veneers were secured in blocks created from auto polymerizing acrylic resin in a custom-made mold, with only the top surface accessible for treatment (Figure 1). The veneers in each group were divided in a random fashion into 2 subgroups (*n* = 12) according to the immersion solutions: black tea (Lipton^®^) and coffee (Nescafe Classic, Nestlé, Switzerland). In each subgroup, the veneers were immersed in the solutions for 3, 5, and 7 days and stored in an incubator (JSR incubator JSGI-150T, Gongju, Korea) at 37 °C. For the tea immersion solution preparation, 2 tea bags were dissolved in 30 mL of boiled water. Meanwhile, for the coffee immersion solution preparation, 2 scoops of coffee were dissolved in 30 mL of boiled water. The immersing mediums were refreshed every other day to prevent bacterial or yeast contamination.

### 2.2. Stain Removal Treatment

The stained veneers in each immersion subgroup were split in a random fashion into two groups, each receiving a different surface treatment: in-office bleaching (*n* = 6) or surface repolishing (*n* = 6). A 40% hydrogen peroxide ~1 mm thick coat (Opalescence^®^ Boost PF 40%, Ultradent Products, Inc., South Jordan, UT, USA) was applied for one hour during the in-office bleaching procedure. After 30 min, the 1 mm thick bleaching coat was refreshed. Afterward, the tested veneers were rinsed thoroughly with water for 30 s and then dried with tissue paper [18]. In the repolishing group (*n* = 6), the outer surface of the tested veneers was polished for 60–80 s using a Soflex^®^ disc sequentially from medium to fine and superfine.

### 2.3. Color Measurement

Measurements of the tested veneers’ L* a* b* values were obtained using a spectrophotometer (LabScan XE^®^, HunterLab, Sunset Hills Road, Reston, VA, USA). The apparatus (0°/45° geometry, D 65 optical sensor, 10° observer) was calibrated using black and white reference tiles with the tri-stimulus values X, Y and Z using values of a white reference tile, (X = 80.84; Y = 85.64; Z = 91.67), as standards. The tested veneers were placed over the spectrophotometer sensor with two different black and white backgrounds. Then, three readings were recorded for black and white backgrounds per tested veneer [19]. The color changes were analyzed using Hunter’s equation [20]:

Δ*E* = [(ΔL*)^2^ + (Δa*)^2^ + (Δb*)^2^]^1/2^

L* = lightness, a* = green (−a) and red (+a) axis, and b* = blue (−b) and yellow (+b) axis.

### 2.4. Surface Roughness

To measure surface roughness (Ra), a noninvasive optical noncontact profilometer (Bruker, Tucson, AZ, USA) was used as detailed in a previous study [21]. This device uses the white light interferometry technique and measures the height differences on the specimen surfaces by utilizing the refractive indices of the white light components [22]. This analysis provides a quantitative assessment of the surface. To determine roughness, a 5× Michelson magnification lens and a Gaussian regression filter were used along with a 1 × 1 mm^2^ field of view and 1× scan speed and a threshold of 4 [23]. The Simple Vision 64 application software (Bruker, Tucson, AZ, USA) was used to convert the data into high-resolution images and control device movements. The system was recalibrated prior to measurements of each subsequent group. The readings were taken from three different areas and presented as an average for each tested veneer to determine the average surface roughness (Ra) in micrometers. The Ra measurements were carried out in compliance with the standardization recommendations of ISO 16610 [24]. In order to ensure the precision of the roughness measurement process, uncertainty and noise, the Bruker Contour GTK profilometer was positioned on a TMC™ Ametek^®^ table with Gimbal Piston air isolators (AMETEK TMC, Peabody, MA, USA) as a standard feature to isolate and cancel out building floor vibrations in nanotechnology. Fine adjustment of the fringes was performed until they were of the optimal size and position (0–15 fringes visible for a VSI measurement). The Gaussian filter was used as a smoothing filter that suppressed noise, using the Gaussian function. A percentage value was set as a threshold, using the automatic feature of the machine, to determine the acceptable signal-to-noise level. Likewise, it prevents saturation of the detector.

### 2.5. Gloss Measurements 

To measure the gloss according to the specifications of ISO 2813 [25], a Novo-Curve gloss meter (Rhopoint Instruments Ltd., East Sussex, UK) was utilized. A gloss meter is a reflectometer that comprises an incandescent light source, a photodetector, and a collimator [26]. The laser beam was directed at a 60° angle onto the surface of the tested veneer, and the intensity of the reflected light was determined by using a photodetector placed in the incident beam’s specular direction. The gloss of a surface was measured by a reflectometer, which calculates the proportion of direct reflected light. The gloss values are presented using gloss units (GUs).

### 2.6. Statistical Analysis

GraphPad Prism 5 software package (GraphPad Software, San Diego, CA, USA) was used to perform the statistical analyses. To analyze the data, normality and homoscedasticity tests were conducted. Then, two-way ANOVA and Tukey’s post hoc method (α = 0.05) were used for all pairwise multiple comparison procedures. 

## 3. Results

The Tukey’s multiple comparison tests showed a statistically significant difference between the Δ*E* values of the tested veneers after artificial aging and their baseline counterparts. 

While at baseline, the Δ*E* values were 8.450 ± 0.192, 7.844 ± 0.341, and 6.889 ± 0.153 for the 3D-printed veneers, C-PRCVs, and E-PRCVs, respectively. On day 7, coffee immersion resulted in Δ*E* values of 10.112 ± 0.141, 9.028 ± 0.220, and 9.795 ± 0.102 for the 3D-printed veneers, C-PRCVs, and E-PRCVs, respectively. 

**Figure 2:**
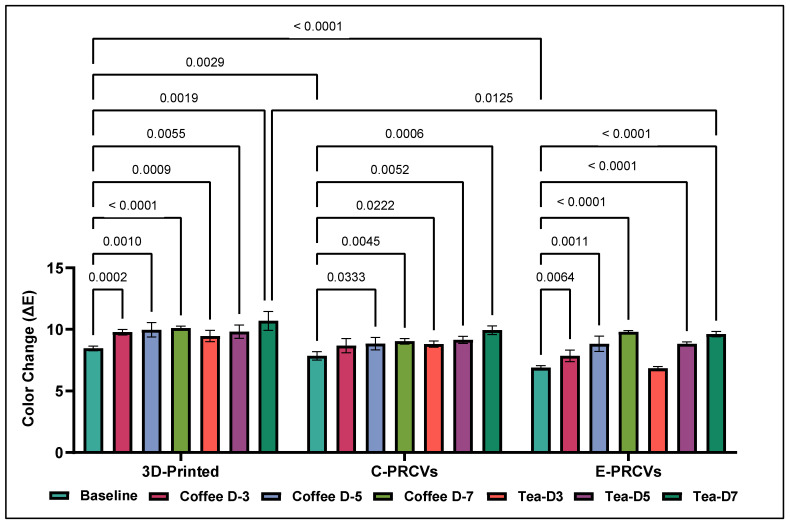
Comparison of the means for color change (Δ*E*) in the 3D-printed veneers, C-PRCVs, and E-PRCVs at the different experimental time points, using the two staining solutions.

Meanwhile, on day 7, immersion in tea resulted in Δ*E* values of 10.689 ± 0.771, 9.926 ± 0.355, and 9.611 ± 0.220 for the 3D-printed veneers, C-PRCVs, and E-PRCVs (Figure 2). 

**Figure 3:**
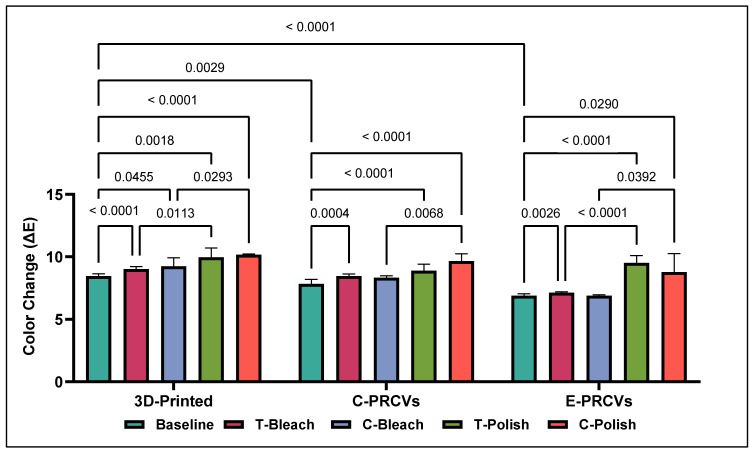
Comparison of the color change (Δ*E*) means after in-office bleaching and surface repolishing of stained 3D-printed veneers, C-PRCVs, and E-PRCVs.

On the other hand, the Δ*E* values after surface treatment with in-office bleaching and surface repolishing were significantly higher than the baseline values for all tested veneers (Figure 3). While surface repolishing resulted in significantly higher Δ*E* values, the in-office bleaching Δ*E* values were comparable to the baseline values, and no significant differences were found in the PRCVS.

**Figure 4:**
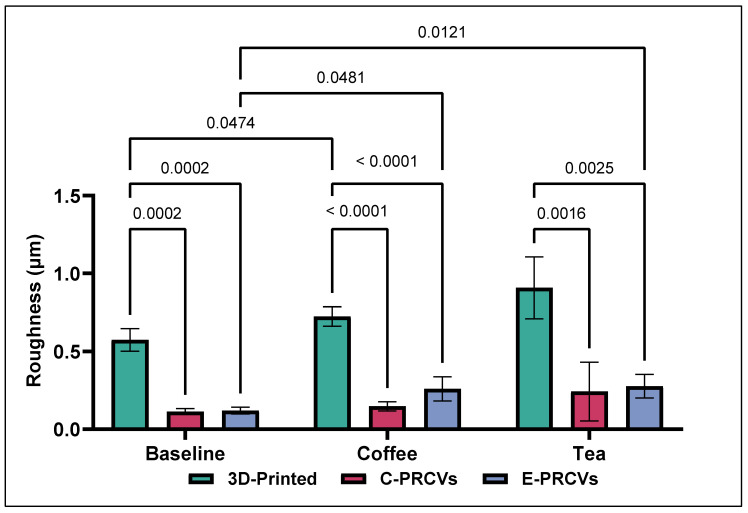
Comparison of the means of surface roughness (Ra) for 3D-printed veneers, C-PRCVs, and E-PRCVs at the different experimental time points, using the two staining agents.

The surface roughness parameter values at baseline and the changes after aging are depicted in Figure 4. The Tukey’s multiple comparisons tests showed statistically significant differences among the 3D-printed and both PRCV systems in surface roughness at baseline *(p =* 0.0002). The 3D-printed veneers demonstrated higher Ra values (0.574 µm ± 0.073), whereas the C-PRCVs and E-PRCVs showed median values of 0.115 µm ± 0.018 and 0.120 µm ± 0.021, respectively. 

Artificial aging by immersion in coffee and tea caused significant changes in Ra values. After immersion in coffee, the 3D-printed veneers showed a significantly higher increase in Ra (0.724 µm ± 0.062) compared to the 3D-printed baseline. Meanwhile, the E-PRCVs showed significantly higher Ra (0.261µm ± 0.078) compared to the C-PRCVs (0.148 µm ± 0.030). However, no statistical difference between the C-PRCVs and E-PRCVS was detected. After immersion in tea, statistically significant differences were found for the pairs of 3D-printed veneers and C-PRCVs (*p =* 0.002) and 3D-printed veneers and E-PRCVs (*p =* 0.003).

**Figure 5:**
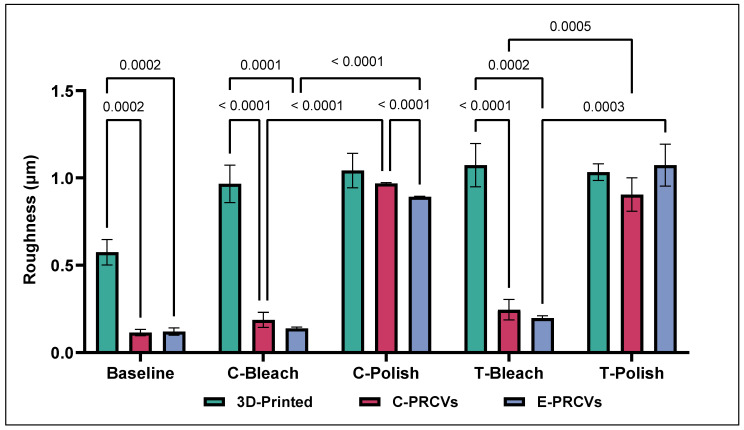
Comparison of the means of surface roughness (Ra) after bleaching and surface repolishing of stained 3D-printed veneers, C-PRCVs, and E-PRCVs.

Further, an increase in surface roughness after surface treatment with in-office bleaching and surface repolishing is depicted in Figure 5. The Tukey’s multiple comparison tests showed statistically significant differences among the 3D-printed and the PRCV veneers in surface roughness after both in-office bleaching and surface repolishing. While the 3D-printed veneer Ra values showed no statistically significant differences regardless of the surface treatment method, in-office bleaching had less effect on the Ra values of both PRCV systems. Surface repolishing resulted in an increase in surface roughness in the C-PRCVs and E-PRCVs compared to baseline (*p* < 0.0001) and (*p* < 0.0003) in coffee and tea, respectively.

**Figure 6:**
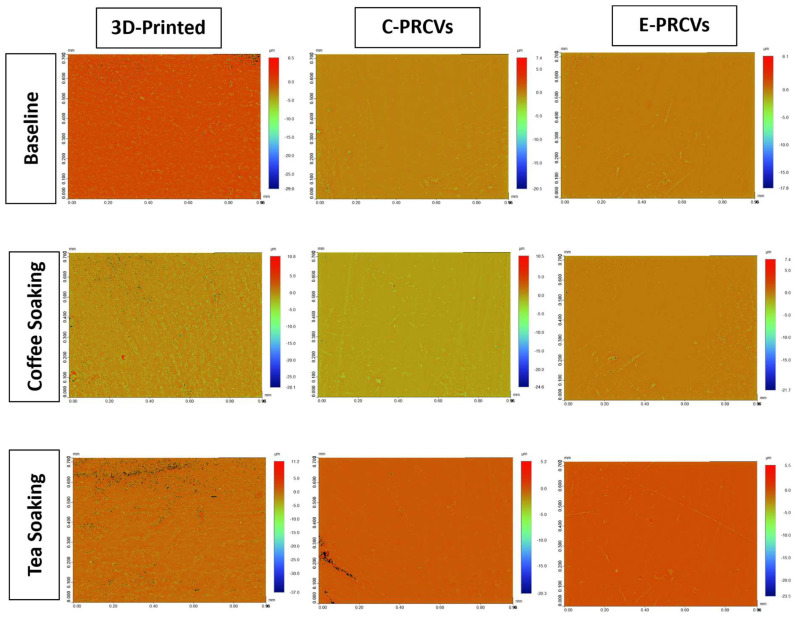
Representative 3D reconstructed images of 3D-printed veneers, C-PRCVs, and E-PRCVs at baseline and after 7 days of staining.

**Figure 7:**
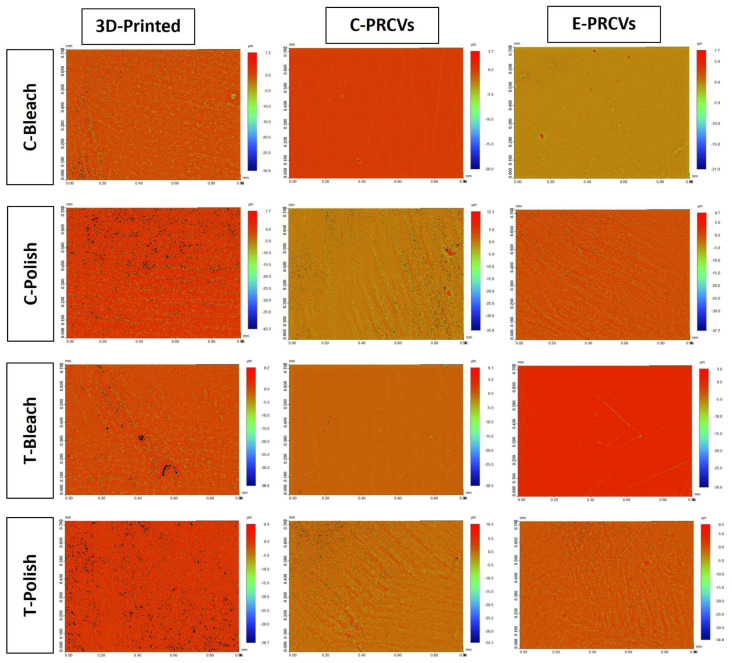
Representative 3D reconstructed images after bleaching and surface repolishing of stained 3D-printed veneers, C-PRCVs, and E-PRCVs.

Figure 6 and Figure 7 show the representative 3D reconstructed images of surface topography obtained for different groups at baseline and after surface treatment using in-office bleaching and surface repolishing. It can be noted that surface treatment with in-office bleaching and surface repolishing presented the roughest surfaces for 3D-printed veneers, while in-office bleaching showed the smoothest surface for the C-PRCVS and E-PRCVs compared to the rough surface produced after surface repolishing.

**Figure 8:**
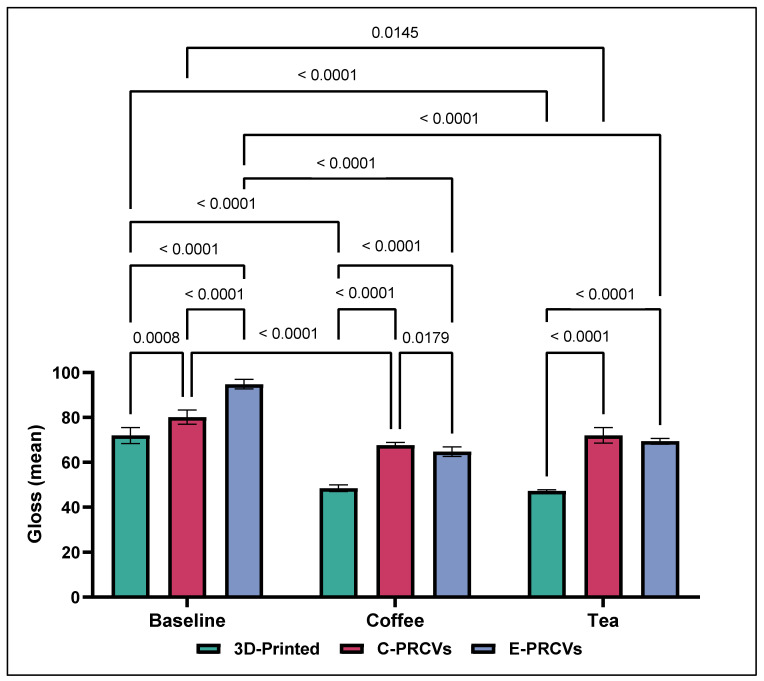
Comparison of the means of surface gloss (GUs) for 3D-printed veneers, C-PRCVs, and E-PRCVs at the different experimental time points, using the two staining agents.

The gloss values at baseline ranged from 71.945 ± 3.620 to 80.095 ± 3.163 and 94.825 ± 2.127 GUs for the 3D-printed veneers, C-PRCVs, and E-PRCVs, respectively (Figure 8). Aging by immersion in coffee resulted in a significant reduction in GUs of the 3D-printed veneers to 48.473 ± 1.505 GUs (*p* < 0.0001) compared to baseline 57.798 ± 1.86 GUs, while that of the C-PRCVs was significantly reduced to 67.637 ± 1.279 GUs (*p* < 0.0001), and that of the E-PRCVs was significantly reduced to 64.777 ± 2.117 GUs (*p* < 0.0001) compared to its counterpart at baseline.

Aging by immersion in tea resulted in a significant reduction in the GUs of 3D-printed veneers to 47.187 ± 0.566 GUs (*p* < 0.0001) compared to baseline, while that of the C-PRCVs was significantly reduced to 72.034 ± 3.531 GUs (*p* < 0.001) and that of the E-PRCVs was significantly reduced to 69.444 ± 1.292 GUs (*p* < 0.0001) compared to its counterpart at baseline. 

**Figure 9:**
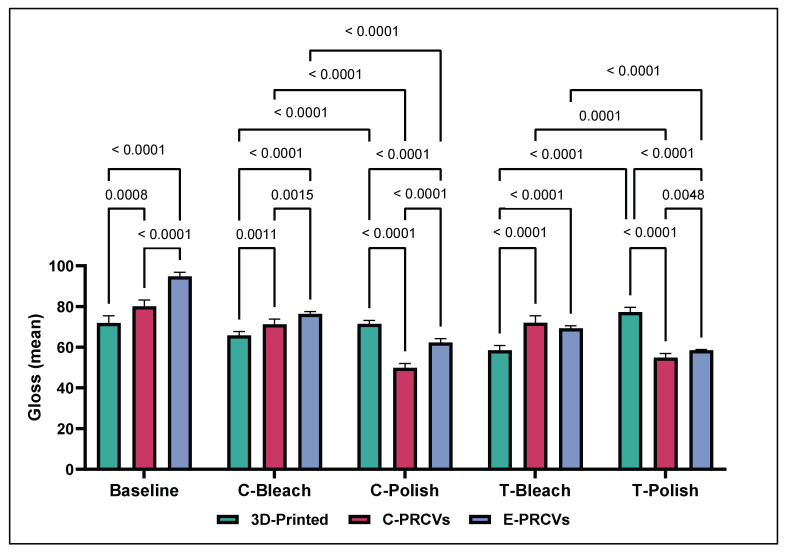
Comparison of the means of surface gloss (GUs) after bleaching and surface repolishing of stained 3D-printed veneers, C-PRCVs, and E-PRCVs.

Statistically significant decreases in gloss values were observed for the C-PRCVs and E-PRCVs after surface repolishing to 49.849 ± 2.307 and 62.327 ± 1.932 GUs for the coffee-immersed samples and to 54.922 ± 2.142 and 58.515 ± 0.426 GUs for the tea-immersed samples, respectively (Figure 9). 

On the contrary, stain removal via in-office bleaching for the coffee-immersed samples resulted in significantly higher gloss values of 71.268 ± 2.623 and 76.362 ± 1.256 compared to those of surface repolishing at 49.849 ± 2.307 and 62,327 ± 1.932, in GUs, for all tested samples of the C-PRCVs and E-PRCVs, respectively. Similarly, stain removal via in-office bleaching for the tea-immersed samples resulted in significantly higher gloss values of 72.034 ± 3.531 and 69.253 ± 1.368 compared to surface repolishing 54.922 ± 2.142 and 58,515 ± 0.426, in GUs, for the C-PRCVs and E-PRCVs, respectively.

Remarkably, the 3D-printed veneers showed significant enhancement in gloss values after surface repolishing (71.479 ± 1.791 and 77.189 ± 2.451) compared to in-office bleaching (65.758 ± 1.994 and 58.581 ± 2.332) in the coffee- and tea-immersed samples, respectively.

## 4. Discussion

Laminate veneers are a minimally invasive treatment option for various dental issues, such as teeth that are resistant to bleaching, require morphological modifications, need gap closure, require minor alignment corrections, or have enamel malformations, fractures, chipping, fluorosis, loss of vertical dimension, attrition, and erosion [1]. Ceramic veneers are traditionally used for such clinical situations. Still, with the recent advances in adhesive systems and nanocomposite resin materials, a less invasive approach is now possible for complex cases. These advancements offer a solution that addresses both aesthetic and functional demands effectively [2]. Moreover, utilizing 3D printing for manufacturing composite resin veneers and PRCVs has many advantages. These minimally invasive techniques help to retain the natural tooth structure [6,8]. Additionally, they require only one appointment and a short time to complete the treatment, making them a convenient on-demand chair-side option. Moreover, they are easily repairable, cost-effective, and a preferred solution to replacement [27]. Importantly, using 3D printing technology for manufacturing dental prostheses reduces waste and enables simultaneous processing of multiple prostheses [11].

Remarkably, the oral cavity can be a challenging environment for dental materials. Rough surfaces and biofilm formation can cause deterioration and staining of dental restoration over time [28]. Composite resin materials are particularly vulnerable to surface damage and discoloration once exposed to an oral environment and extrinsic stains [12]. Common daily used agents such as coffee and tea may significantly alter the value, chroma, or hue of natural teeth or dental restorative materials [29]. A previous study reported that one day of immersion in discoloring agents corresponds to 1 month of clinical function [30,31]. Here, a seven-day immersion period was chosen, equal to approximately seven months of clinical aging (24 h of staining in vitro corresponds to about 1 month in vivo). Nevertheless, after 7 days of artificial aging, all noticeable color changes Δ*E* fell within the range of (coffee = 10.112 ± 0.141, tea = 10.689 ± 0.771), (coffee = 9.028 ± 0.220, tea = 9.926 ± 0.355) and (coffee = 9.795 ± 0.102, tea = 9.611 ± 0.220) for the 3D-printed veneers, C-PRCVs, and E-PRCVs, respectively. Notably, all tested veneers showed significant color changes compared to the values prior to artificial aging, whereas the highest values were recorded for the 3D-printed veneers. That being said, before artificial aging, the PRCVs displayed lower surface roughness than that of the 3D-printed veneers, implying that a lower level of surface roughness protects the PRCVs from further discoloration. However, over time, artificial aging with staining tests increased the discoloration tendency significantly in all tested veneers. The results support the notion that those differences in discoloration susceptibility between the 3D-printed and PRCVs could be due to differences in each system’s inherent structure and material composition. In agreement with this is the fact that the color change in a resin material dramatically depends on the composition of the resin and filler content [32]. Resin-based materials with lower filler content absorb more water at the resin–filler interface, leading to hydrolytic degradation of the filler and ultimately leading to a greater susceptibility to staining [33,34]. Particularly, discoloring pigments are carried primarily by water; water penetration is more likely to occur in the resin matrix of composite veneers [35]. Thus, discoloring particles have the ability to seep into the resin matrix and gather inside the composite, ultimately leading to discoloration [36].

Our results reject the first null hypothesis that there is no difference in discoloration tendency among the tested veneers. Furthermore, the results are consistent with those of Stawarczyk et al. who also observed variations in the discoloration of different composite veneering materials when exposed to staining solutions [37]. Moreover, Alharbi et al. found that 3D-printed veneers exhibited a higher tendency to discolor agents compared to milled composite-based veneers [18]. The color changes observed in this study for both the 3D-printed and milled composite-based veneers upon immersion in tea and coffee are completely unacceptable for permanent long-term use, in accordance with a previous study on 3D-printed and milled materials [18]. The results of the current in vitro experiment revealed that the tested 3D-printed material exhibited a higher color change value.

Remarkably, the manufacturing process significantly impacts filler loading and the surface properties of the 3D-printed and PRCVs. The E-PRCVs are created using high-pressure molding and heat-curing techniques, which are then followed by laser surface vitrification. The C-PRCVs, on the other hand, are molded and light-cured without any special tempering or surface treatment. Hence, a more durable and glossier surface can be produced by incorporating surface laser vitrification or glazing of the outer surface. It has been suggested that laser treatment can result in smooth surface vitrification and increased hardness, wear resistance, and color stability. In contrast, the low filler-loading of the current 3D printing technology and the lack of post-processing surface modifications significantly affect the optical properties of the 3D-printed veneers. Furthermore, the 3D-printed material is more likely to get stained due to the manufacturing process that involves stacking multiple layers on top of each other. The possibility of incomplete polymerization at the interface of these layers, along with the presence of microporosities and residual monomers, can lead to a higher chance of discoloration in printed materials [18,38].

Beyond the choice of material, the surface quality can affect plaque accumulation and restoration discoloration [18]. Furthermore, composite-based materials tend to absorb more water, leading to the breakdown of their organic matrix and resulting in rougher surfaces [35]. Notably, coffee and tea cause color changes due to low polarity molecules that penetrate the resin matrix’s deeper layers [39]. In addition to causing matrix degradation and the absorption/adsorption of colorants, they also increase the surface roughness of restorative materials due to the presence of tannins [30,40]. In this study, at baseline, the PRCVs had significantly lower Ra values than the 3D-printed veneers. Notably, the baseline surface quality of the E-PRCVs displayed a notable reduction in surface roughness compared to the C-PRCVs. The manufacturer of the E-PRCVs claims to have developed a tempering process for the final surface finish. This might be the reason for the improved surface quality at baseline.

The PRCVs had a rougher surface after surface treatment compared to the 3D-printed veneers. After in-office bleaching, the surface roughness of the tested composite veneers returned to values comparable to baseline values of 0.115 ± 0.018 µm and 0.121 ± 0.021 µm for the C-PRCVs and E-PRCVs, respectively. Those values were within the range of 0.187 ± 0.043 µm and 0.139 ± 0.008 µm for the coffee-stained veneers and 0.245 ± 0.058 µm and 0.198 ± 0.014 µm for the tea-stained veneers. However, values fell above the acceptable threshold after surface repolishing, where 0.25 to 0.50 µm is the threshold the human tongue can perceive [41]. This indicates that repolishing the surface leads to mechanical removal of dislodged filler particles, resulting in a rough surface.

It is worth noting that this study only used the Sof-Lex polishing system. Hence, it is necessary to explore the applications of different tools and polishing systems. Moreover, during the polishing process, changes occur in the surface layer of the material, such as selective extraction of filler particles from the mass of material or wiping the resin, which leaves protruding filler particles [1]. Therefore, it is important to compare the hardness and chemical composition before and after polishing to gain better understanding of the process. Previous studies of the effect of different polishing systems indicated variable effect on surface roughness and color stability. Freitas et al. found a strong correlation between color stability and the finishing/polishing procedure, where higher surface roughness values correspond to greater color differences [42]. 

On the other hand, gloss is an optical phenomenon that demonstrates a surface’s ability to reflect directed light and considerably contributes to its aesthetic properties [43]. Filler loading, size distribution, and refractive index are all considered significant variables in a resin composite [44]. According to the ASTM D523 standard, values (>70 GUs) are regarded as high-gloss surfaces [45]. After artificial aging, these values dropped to 48.47 ± 0.5 and 47.187 ± for the 3D-printed veneers. Moreover, the coffee-stained veneers reached values of 67.637 ± 1.279 and 64.777 ± 2.117 and the tea-stained veneers 72.034 ± 3.531 and 69.44 ± 1.292 in the C-PRCVs and E-PRCVs, respectively. These findings reject the second null hypothesis that there is no difference in surface roughness and gloss retention between the different veneer materials.

Although increased color changes, surface roughness, and reduced gloss retention can compromise aesthetic outcomes, composite resin veneers are easily reparable, prolonging restoration durability. Therefore, bleaching and surface repolishing are proposed for stain removal. While bleaching tends to be more effective in removing stains, the E values in this experiment failed to return to the baseline values, indicating that the stains penetrated the matrix rather than being adsorbed on the veneer’s surface. This is in agreement with the findings of Türkün et al. who reported that polishing was less effective than bleaching due to the deeper penetration of stains [17].

Remarkably, bleaching and surface repolishing increased surface roughness in both the 3D-printed veneers and PRCVs. It has been reported that the abrasives used must be harder than the composite filler to ensure successful repolishing of resin composite work [34]. If the polishing discs are not harder than the filler, they will only remove the soft resin matrix and leave the filler particles protruding from the surface [34]. Furthermore, bleaching can lead to the selective softening of the resin matrix and dislodging of filler particles [46]. Additionally, bleaching agent free radicals impact the resin–filler interface and cause filler–matrix debonding [33,46]. This is in agreement with the findings of Abd Elhamid et al. who reported that bleaching with carbamide peroxide had an unfavorable effect on the surface quality of composite resin materials [33]. To that end, the third null hypothesis was rejected: bleaching and surface repolishing do not affect the color changes, surface roughness, and gloss retention of the tested veneer materials.

Although the past literature has pointed out the potential discoloration caused by different staining solutions and provided modalities for stain removal, this specific study delved for the first time into our knowledge of the impact of artificial aging on the discoloration of a novel 3D-printed veneer material in comparison to two established prefabricated composite veneer systems. Yet, to provide more comprehensive understanding of the material surface properties, further research is necessary with different staining agents. Moreover, a limitation of the current study was that the staining solutions were used in static conditions. Therefore, future investigations should involve cycling specimens in staining solutions at different temperatures to better simulate the oral environment, considering that various beverages and foods are consumed at varying temperatures. Further clinical studies are recommended to characterize other intraoral factors contributing to the surface and optical properties of 3D-printed resin composite veneers. Moreover, studies to advance material processing for 3D printing can be seen as a milestone in dentistry. This will significantly contribute to composite application and help a larger number of patients to receive affordable and conservative aesthetic restorations.

## 5. Conclusions

Within the limitations of this in vitro study, the following conclusions can be drawn:

1. Veneers manufactured using the 3D printing technique are vulnerable to discoloration and are significantly affected by artificial aging in a staining solution compared to the PRCVs.
2. Coffee and tea staining have a deleterious effect on the color, surface gloss, and surface roughness of all tested indirect composite veneers despite manufacturing techniques.
3. The efficacy of stain removal was higher with an in-office bleaching technique compared to surface polishing in the PRCVs, while in-office bleaching and surface polishing showed comparable effects in the 3D-printed veneers.
4. Veneer production using 3D printing provides cost-effective, time-efficient and on-demand solutions. However, material processing for 3D printing is crucial for long-term longevity.

## Figures and Tables

**Table 1:** Compositions of tested veneers.

Material	Composition
Irix Max DWS ^a^	Photosensitive ceramic-filled hybrid composite material (42% of ceramic in weight)
Componeer Brilliant ^b^	Organic matrix: BISGMA, TEGDMA Photoinitiator and co-initiators Inorganic filler size 0.02 to 2.5 µm (80 wt%)
Edelweiss Direct Veneers ^c^	Highly filled nanohybrid composite filling material (83 wt%)

^a^ DWS; ^b^ Coltene Whaledent; ^c^ Edelweiss Dentistry. Source: DWS, Componeer, and Edelweiss brochures.

## Data Availability

The data is available on request from the corresponding author.

## Abbreviations

PRCVs	Prefabricated resin composite veneers
C-PRCVs	Colten prefabricated resin composite veneers
E-PRCVs	Edelweiss prefabricated resin composite veneers
T-Bleach	Bleached tea-stained veneers
C-Bleach	Bleached coffee-stained veneers
T-Polish	Polished tea-stained veneers
C-Polish	Polished coffee-stained veneers
GU	Gloss unit
AM	Additive manufacturing
TSLA	Tilted stereolithography
CAD	Computer-aided design
